# Differences in Human Plasma Protein Interactions between Various Polymersomes and Stealth Liposomes as Observed by Fluorescence Correlation Spectroscopy

**DOI:** 10.1002/mabi.202200424

**Published:** 2022-11-29

**Authors:** Adrian Najer, Omar Rifaie-Graham, Jonathan Yeow, Christopher Adrianus, Mohamed Chami, Molly M. Stevens

**Affiliations:** Department of Materials Department of Bioengineering and Institute of Biomedical Engineering Imperial College London London SW7 2AZ, UK; Department of Materials Department of Bioengineering and Institute of Biomedical Engineering Imperial College London London SW7 2AZ, UK; Department of Materials Department of Bioengineering and Institute of Biomedical Engineering Imperial College London London SW7 2AZ, UK; Department of Materials Department of Bioengineering and Institute of Biomedical Engineering Imperial College London London SW7 2AZ, UK; BioEM lab, Biozentrum, University of Basel, Mattenstrasse 26, Basel 4058, Switzerland; Department of Materials Department of Bioengineering and Institute of Biomedical Engineering Imperial College London London SW7 2AZ, UK

**Keywords:** fluorescence correlation spectroscopy, human plasma protein fouling, liposome, polymersome, protein corona

## Abstract

A significant factor hindering the clinical translation of polymersomes as vesicular nanocarriers is the limited availability of comparative studies detailing their interaction with blood plasma proteins compared to liposomes. Here, polymersomes are self-assembled via film rehydration, solvent exchange, and polymerization-induced self-assembly using five different block copolymers. The hydrophilic blocks are composed of anti-fouling polymers, poly(ethylene glycol) (PEG) or poly(2-methyl-2-oxazoline) (PMOXA), and all the data is benchmarked to PEGylated “stealth” liposomes. High colloidal stability in human plasma (HP) is confirmed for all but two tested nanovesicles. In situ fluorescence correlation spectroscopy measurements are then performed after incubating unlabeled nanovesicles with fluorescently labeled HP or the specific labeled plasma proteins, human serum albumin, and clusterin (apolipoprotein J). The binding of HP to PMOXA-polymersomes could explain their relatively short circulation times found previously. In contrast, PEGylated liposomes also interact with HP but accumulate high levels of clusterin, providing them with their known prolonged circulation time. The absence of significant protein binding for most PEG-polymersomes indicates mechanistic differences in protein interactions and associated downstream effects, such as cell uptake and circulation time, compared to PEGylated liposomes. These are key observations for bringing polymersomes closer to clinical translation and highlighting the importance of such comparative studies.

## 1 Introduction

The interaction of blood plasma proteins with nanoparticles, also commonly referred to as protein fouling or protein corona formation, is a key factor determining cell interactions, targeting ability, non-specific organ uptake, and blood circulation time.^[1,2]^ There is evidence that protein fouling on nanomedicines plays a role in humans, as the presence of a protein corona was confirmed on PEGylated liposomes after injection and recovery from patients.^[3]^ In general, extensive protein fouling on nanoparticles should be avoided, because it often leads to fast macrophage uptake and short circulation times.^[4–6]^ However, the adsorption of certain specific types of proteins can be beneficial in terms of delaying uptake and prolonging blood residence time.^[7]^

Blood plasma proteins can be categorized into opsonins and dysopsonins. Opsonins generally mediate rapid cell uptake and elimination of nanoparticles from the bloodstream while dysopsonins generate the opposite effect.^[8]^ To allow for longer circulation periods, nanoparticle surfaces should be engineered to repel opsonins and attract dysopsonins such as human serum albumin (HSA) and clusterin (also known as apolipoprotein J).^[9]^ It was recently demonstrated that clusterin accumulation on PEGylated polystyrene nanoparticles was crucial to allow for their prolonged blood residence^[7]^ Clusterin binding has since been observed for several other types of long-circulating nanoparticles^[10–12]^ but it was not necessary for obtaining long circulation time in all cases.^[13]^ For instance, poly(ethylene glycol) (PEG) grafting density seems to be an important factor influencing clusterin binding. However, there is conflicting data showing the influence of low and high PEG grafting density levels on the clusterin binding efficiency^[14,15]^ This highlights the challenge of generalizing results from existing literature and suggests the need to evaluate each nanoparticle formulation during its development process.

Nanoscale membrane-based vesicular nanoparticles (nanovesicles) have received considerable attention because they can simultaneously encapsulate hydrophilic cargo, such as enzymes, in their inner aqueous lumen, as well as hydrophobic cargo in their membrane. Two common types of vesiclebased nanoparticles are liposomes, composed of ensembles of lipids, and polymersomes, composed of amphiphilic block copolymers.^[16]^ Liposomes were the first clinically approved nanocarriers for drug delivery and are still the most commonly employed systems. However, due to the high chemical versatility of polymer architectures, polymersomes can be engineered to present tunable chemical and mechanical stability, as well as stimuli-responsiveness.^[17]^ Thus, polymersomes represent a unique platform to target applications beyond drug delivery, such as nanoreactors and artificial organelles, in immunotherapy and biosensing.^[18–21]^ To enable low protein fouling, nanovesicles generally present highly hydrated hydrophilic polymers on their surface. In this regard, PEG has been employed as the gold standard polymer of choice and is present in commercialized formulations, such as Doxil, composed of PEG-modified (PE-Gylated) “stealth” liposomes. However, due to the emergence of anti-PEG antibodies, the development of nanovesicles with alternative hydrophilic polymers, such as poly(2-oxazoline)s, including poly(2-methyl-2-oxazoline) (PMOXA), is of paramount importance.^[22,23]^

PEGylated liposomes generally present low protein fouling, resulting in a delay in their ability to be uptaken by cells.^[24]^ This property of PEGylated liposomes is also influenced by the lipid bilayer phase behavior: gel-phase liposome membranes bind more protein than liquid-phase liposomes.^[25]^ Clusterin has also been found in the protein coronas forming around PEGylated liposomes.^[11]^ In contrast, only a few polymersome-protein fouling studies are available to date, showing specific formulation properties rather than generalizable trends.^[18,20]^ For instance, polymersomes composed of methoxy-terminated poly(ethylene glycol)-*b*-poly(propylene sulfide) (PEG-*b*-PPS) were found to interact with more protein units than micelles (non-hollow lamellar ensembles with hydrophobic core) and filomicelles made from the same copolymer after 24 h.^[26]^ Another study showed that polymersomes composed of poly(2-methyl-2-oxazoline)-*b*-poly(dimethylsiloxane)-*b*-poly(2-methyl-2-oxazoline)(PMOXA-*b*-PDMS-*b*-PMOXA) bound to serum proteins including clusterin.^[27]^ In contrast, polymersomes derived from polymerization-induced self-assembly (PISA) presenting a hydrophilic block of poly(2-(methacryloyloxy)ethylphosphorylcholine (PMPC) revealed low protein binding.^[28]^

The downstream effects of nanovesicle-protein interactions are multifold. For example, polymersomes composed of PEG-*b*-PSS have been observed to bind to albumin. Depending on the end group of the PEG block, albumin denaturation was produced playing a role in the ability of the polymersomes to be taken up by macrophages.^[29]^ The formation of protein corona on other polymersomes has also been observed to reduce toxicity.^[30]^ In addition, several^[31,32]^ but not all^[33]^ studies showed similar or even longer circulation times for PEG-derived polymersomes (with hydrophobic blocks composed of poly(butadiene) (PEG-*b*-PBD) and poly(D,L-lactide) (PEG-*b*-PDLLA)) compared to PEGylated liposomes. In contrast, PMOXA-polymersomes have been shown to bind to fetal bovine serum (FBS) proteins and revealed worse circulation time in the zebrafish embryo model when compared to PEG-polymersomes and PEGylated liposomes.^[27]^ Moreover, poly(ethylene glycol)-*b*-poly(2-hydroxypropylmethacrylate) (PEG-*b*-PHPMA)-based polymersomes derived from PISA have been shown to protect encapsulated enzymes from proteases and indicating long circulation time (although quantitative results were not reported).^[34,35]^ Overall, comprehensive studies comparing polymersomes and liposomes in terms of protein corona formation and the associated downstream effects are limited in the scientific literature and are one of the major factors hindering the clinical translation of polymersomes.^[18]^

It should be noted that studying protein fouling on liposomes and polymersomes is challenging with standard techniques that require separation from unbound proteins.^[11]^ Hence, employing techniques that detect protein corona formation in situ would be highly beneficial. In particular, fluorescence correlation spectroscopy (FCS) is an ideally suited method for nanoparticle characterization, including analysis of protein fouling, as it can be performed with non-purified samples and only requires the labeling of plasma or individual proteins with fluorophores.^[36]^ In FCS, fluorescence intensity fluctuations due to Brownian motion are recorded using a confocal setup, the data is autocorrelated and fitted to provide information on diffusion time, hence, hydrodynamic size, concentration, and brightness per nanoparticle.^[37]^ Since its establishment, FCS has proven to be a useful technique to study protein interactions with various nanoparticles^[25,38–40]^ including polymersomes.^[27,41]^ Since FCS is disproportionately biased toward brighter, slower diffusing species,^[42]^ putting the fluorescent labels on the fast-diffusing species (human plasma, HP) creates a setup where even little protein binding will be detected.

Here, we present a broad comparison of protein fouling on a library of polymersomes, formed via three common polymersome formation techniques. We explored interactions of these polymersomes with HP and individual proteins (HSA and clusterin) and compared them to PEGylated liposomes. We first confirmed the stability of most nanovesicles when incubated with 10 v/v% HP. This library of polymersomes was then studied for protein binding using in situ FCS. Thus, we incubated either: randomly fluorescently labeled HP with Oregon Green 488 (HP-OG488), HSA-OG488, or clusterin-OG488 in the presence of nanovesicles and monitored the appearance of diffusion times corresponding to nanoparticles, which indicated protein binding. This analysis allowed the detection of small differences in protein fouling on the various types of polymersomes compared to PEGylated liposomes. The main difference found was that the clusterin-binding efficiency was lower on polymersomes compared to PEGylated liposomes. The results suggest that different mechanisms are involved in the long circulation time of polymersomes compared to PEGylated liposomes, representing a key observation for bringing polymersomes closer to clinical translation.

## 2 Results and Discussion

### 2.1 Synthesis and Characterization of the Nanovesicle Library

**Scheme 1:**
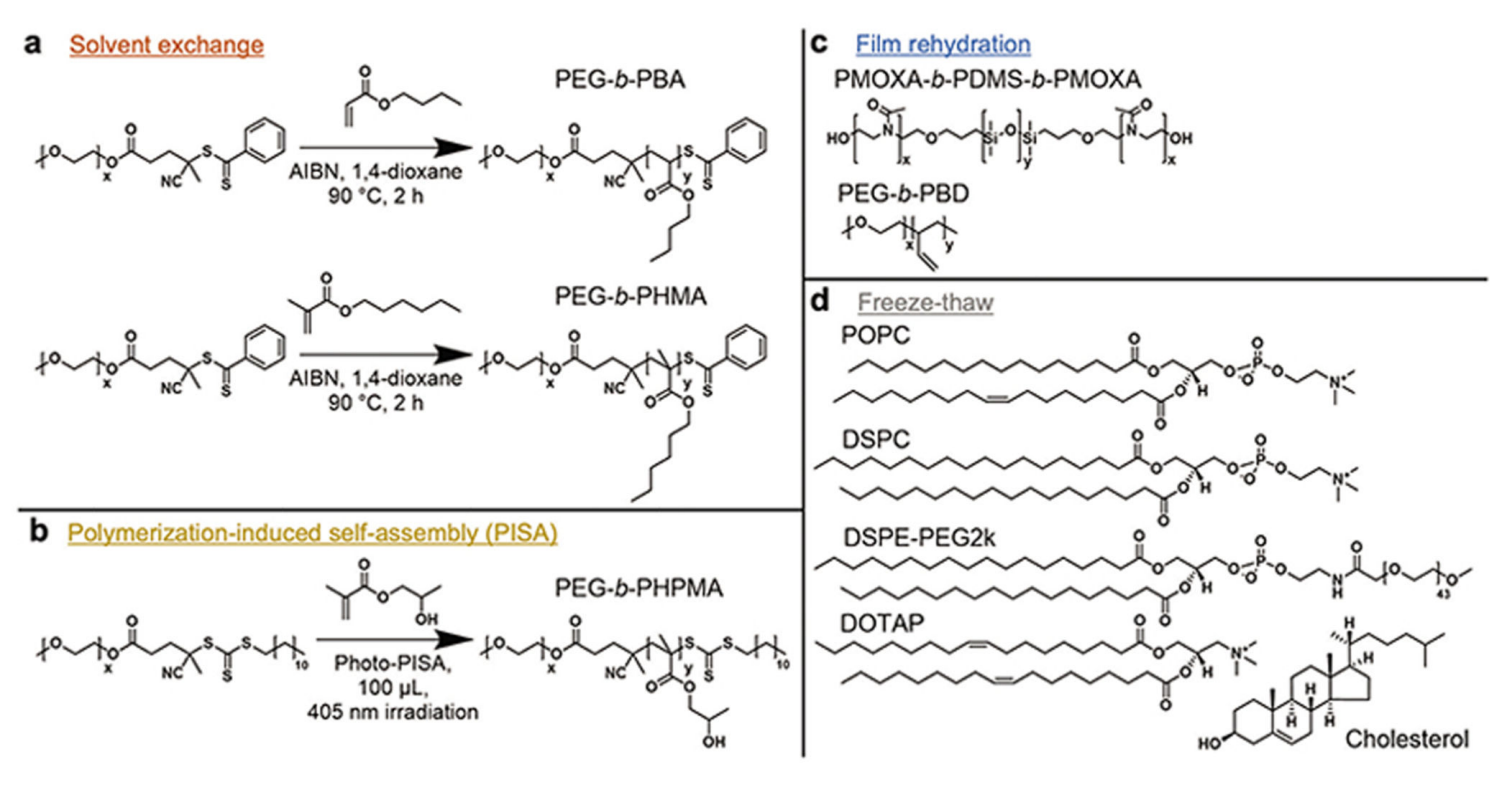
Schematics of copolymers, lipids, and self-assembly methods used. a) Synthesis of PEG-*b*-PBA and PEG-*b*-PHMA, which were subsequently self-assembled by the solvent exchange method. b) Photo-PISA synthesis of PEG-*b*-PHPMA polymersomes. c) Commercial PMOXA-*b*-PDMS-*b*-PMOXA and PEG-*b*-PBD were self-assembled through film rehydration. d) Liposomes were assembled through a freeze-thaw technique using either POPC-Cholesterol-DSPE-PEG2k, DSPC-Cholesterol-DSPE-PEG2k, or POPC-Cholesterol-DOTAP lipid mixtures. The degrees of polymerization (DPs, *x* and *y* in schematics) are summarized in Table 1.

To provide a broad overview of HP protein interactions with a library of polymersome compositions compared to lipidic systems, we synthesized block copolymers and self-assembled poly-mersomes through three widely established methods:^[19]^ film rehydration, solvent exchange, and photo-PISA (Scheme 1 and Table 1). To enable consistency with past and future research literature, we targeted copolymer systems that have been widely employed in the polymersome field previously (see subsequent references).

Two copolymers were synthesized and assembled through solvent exchanged:^[43–46]^ poly(ethylene glycol)-*b*-poly(butyl acrylate) (PEG-*b*-PBA), and poly(ethylene glycol)-*b*-poly(hexyl methacrylate) (PEG-*b*-PHMA) (Figure S1, Supporting Information). Commercial PEG-*b*-PBD and PMOXA-*b*-PDMS-*b*-PMOXA were used for polymersome formation by film rehydration.^[27,31,33]^ Finally, polymersomes composed of PEG-*b*-PHPMA were synthesized by photo-PISA in microtiter plates as described in our recent paper (Figure S1, Supporting Information).^[34,35]^ Gel-phase PEGy-lated liposomes were formed using the formulation employed in commercialized Doxil (DSPC-Cholesterol-DSPE-PEG2k).^[47]^ Additionally, a liquid-phase version of the same liposomes was formed by substituting DSPC with POPC as the main vesicleforming lipid. DOTAP-containing liposomes without DSPE-PEG2k served as a positive control and are expected to bind large amounts of plasma proteins through electrostatic interactions.

For simplicity, the sample names are abbreviated in the next sections as follows: PMOXA (PMOXA-*b*-PDMS-*b*-PMOXA-1), PMOXA-mix (50 mol% mixture of the two copolymers PMOXA-*b*-PDMS-*b*-PMOXA-1 and PMOXA-*b*-PDMS-*b*-PMOXA-2), PEG-PBD (PEG-*b*-PBD), PEG-PBA (PEG-*b*-PBA), PEG-PHMA (PEG-*b*-PHMA), PISA-PEG (PEG-*b*-PHPMA via photo-PISA), DSPC-PEG (Doxil-mimicking stealth liposomes), POPC-PEG (liquid-phase version of DSPC-PEG), and DOTAP (positively charged control liposomes).

**Figure 1:**
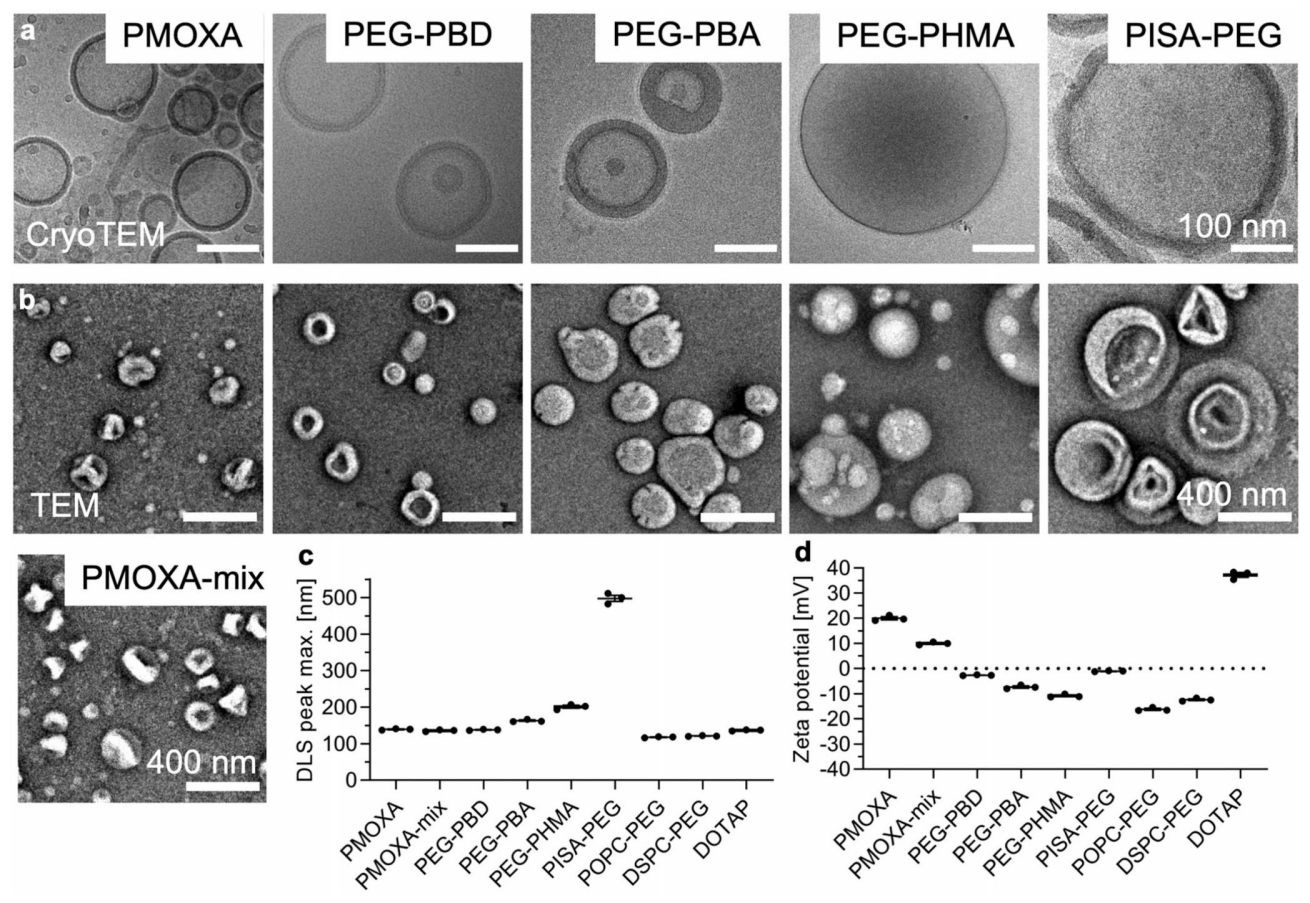
Polymersome and liposome characterization. a) Cryo-TEM images. Scale bars 100 nm. More images can be found in Figures S2 and S3, Supporting Information. b) Negatively stained TEM images. Scale bars 400 nm. c) DLS peak maximum values from intensity distributions in PBS directly after the formation of all nanovesicles used herein (mean of technical triplicates; for distributions, please see Figure 2, PBS t0). d) Zeta potential values for all nanovesicles used herein (mean of technical triplicates, the distribution curves are shown in Figure S4, Supporting Information).

The two synthesized block copolymers PEG-PBA and PEG-PHMA were analyzed by ^1^H NMR and gel permeation chromatography (GPC) (Figure S1, Supporting Information), which confirmed the successful synthesis of the desired copolymers. After self-assembly, all the polymersome samples were characterized by negatively stained transmission electron microscopy (TEM), cryogenic-TEM (cryo-TEM), dynamic light scattering (DLS), and zeta potential measurements. Vesicular morphology was found for all polymersome samples (Figure 1 and Figures S2 and S3, Supporting Information) with diameters ranging from about 100–500 nm.

The TEM and cryo-TEM images, DLS, and zeta-potential values are all in agreement with the previous literature cited above for all the different block copolymers. The samples were intentionally prepared as in existing literature (see citations in the above paragraphs) for the respective block copolymers to ensure comparative data. Extrusion was not performed on poly-mersomes that are not routinely extruded in the field (PEG-PBA, PEG-PHMA, PISA-PEG). Thus, polymersomes with different size distributions were obtained. Overall, this initial analysis of morphology, size, and zeta potential confirmed the successful formation of polymersomes composed of five chemically different block copolymers employing three common nanovesicle formation techniques.

### 2.2 Antifouling Effect of Polymersomes versus Liposomes

Avoiding high levels of non-specific protein accumulation on nanomedicines is of key importance to enable their successful activity in vivo.^[4–6]^ In the most extreme cases, protein binding induces nanoparticle aggregation, causing rapid uptake in the liver and spleen, which ultimately reduces their circulation time in blood. As protein corona formation on nanoparticles has been observed to be species-specific, research studies should be carried out employing human proteins^[48,49]^ and HP rather than serum.^[50]^ In addition, purification-free in situ methods, such as FCS performed in this work, are advantageous for the characterization of protein fouling on light, water-filled nanoparticles such as our nanovesicles.

**Figure 2:**
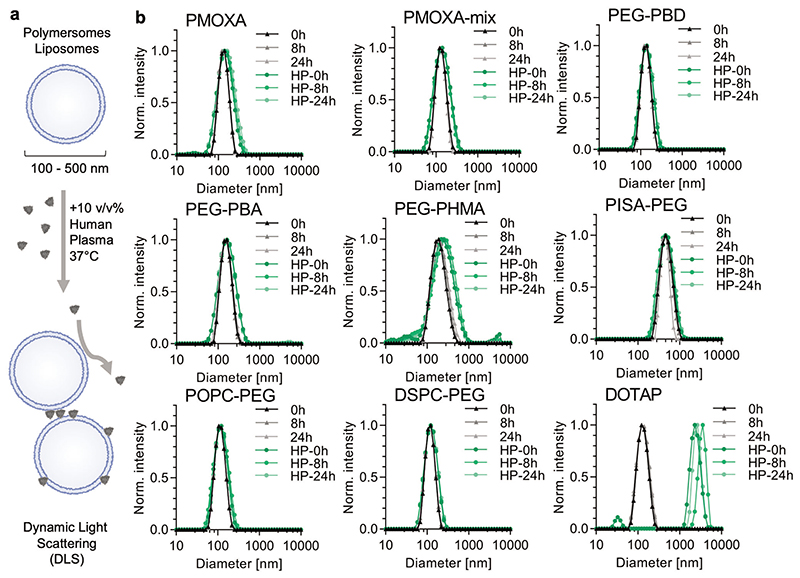
Polymersome and liposome stability in PBS +/− 10 v/v% human plasma (HP) measured by dynamic light scattering (DLS). a) Schematic of incubation procedure. b) DLS intensity distributions for all the tested polymersomes and liposomes after incubation in either PBS (black-grey curves) or PBS + 10 v/v% human plasma (green curves) at 37 °C for 0, 8, and 24 h (mean of technical triplicates, intensity values).

Initially, DLS measurements were employed to comprehensively assess details of the bulk sample stability of the nanovesicles in PBS +/− 10 v/v% HP (Figure 2). All the studied nanovesicles revealed stable hydrodynamic sizes when they were only incubated in PBS. In HP, differences in colloidal stability were more readily observed with the highly positively charged DOTAP liposomes aggregating, which served as an ideal positive control. This was expected due to the anionic nature of most plasma proteins which facilitate electrostatic interactions and cause interconnection between DOTAP-liposomes. In contrast, PEGylated liposomes and most polymersome samples, except PEG-PHMA, remained colloidally stable even after 24 h incubation at 10 v/v% HP. The thinner membrane of PEG-PHMA polymersomes compared to all the other polymersomes (see cryo-TEM images in Figure 1) is one possible explanation for their lower stability in highly concentrated proteinaceous environments.

The colloidal stability of nanovesicles is crucial to enable their potential application in nanomedicine. However, nanovesicle formulations can be stable in blood plasma while interacting with proteins. Thus, we employed in situ FCS to monitor the interaction of randomly labeled HP, referred to as HP-OG488, with unlabeled nanovesicles at a physiological temperature of 37 °C. Initially, using this complete plasma mixture of proteins rather than individual proteins allowed for protein–protein interactions to occur on the nanoparticles in addition to the protein–nanoparticle interactions.

**Figure 3:**
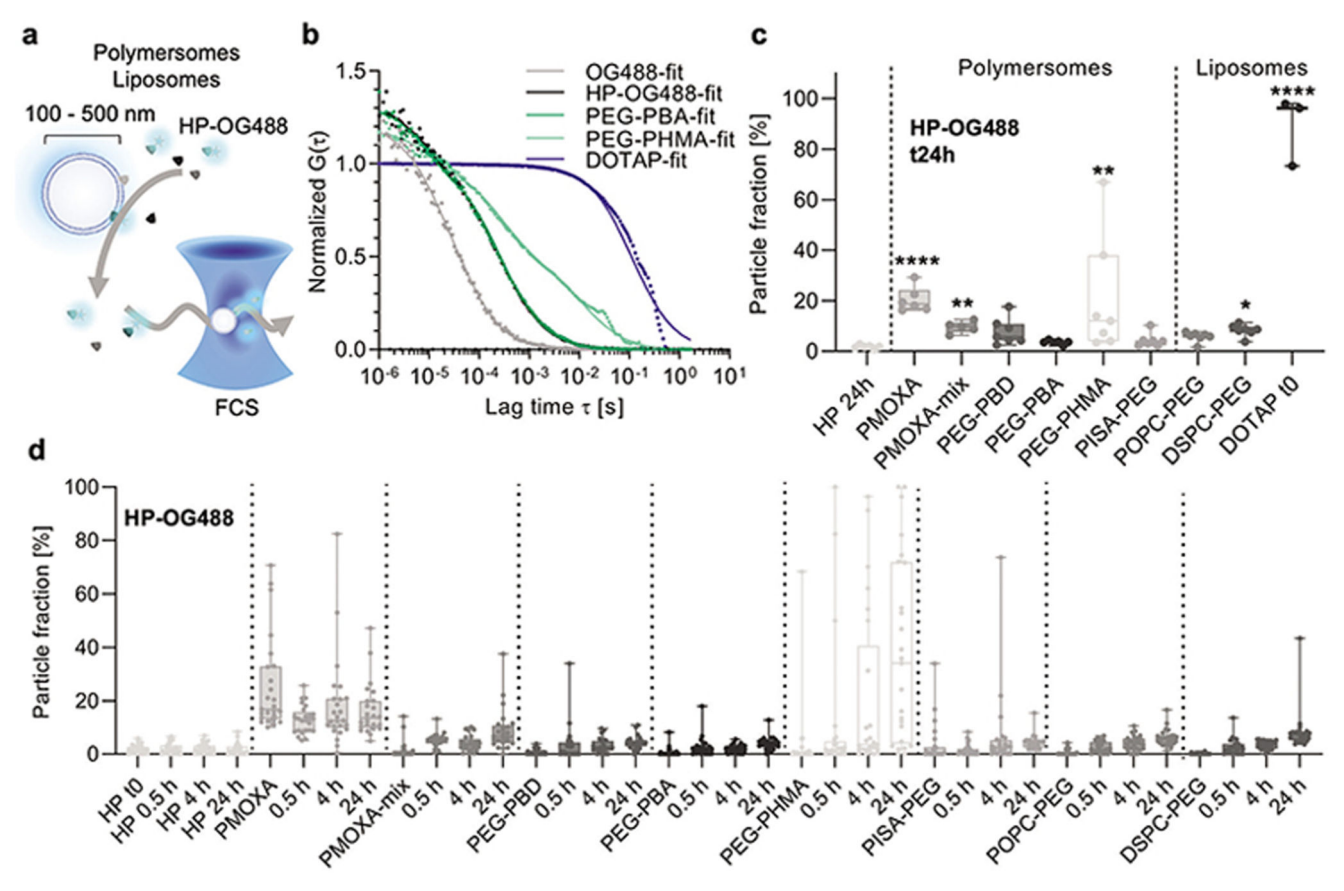
Interaction of randomly labeled human plasma (HP-OG488) with unlabeled polymersome and liposome samples measured by in situ FCS. a) Schematic representation of the FCS experiment. b) Normalized FCS autocorrelation curves for the time point at 24 h (the symbols represent raw data, the solid lines are two-component fits, the average curve of *n* = 25 technical repeats, total 125 s traces). c) Particle fractions obtained from two-component fits of FCS autocorrelation curves as shown in (b) (see Materials and Methods in Supporting Information) for unlabeled polymersomes or liposomes mixed with HP-OG488 after 24 h incubation at 37 °C. The data reveals the extent of protein binding to the nanovesicle surfaces (*N* ≥ 3 independent experiments, each dot representing average values from *n* = 25 technical repeats, as shown in (d)). The high particle fractions represent high protein binding, while low particle fractions represent low protein binding. For the DOTAP nanovesicles, the time point at 0 h is shown, since the addition of HP-OG488 resulted in severe aggregation and sedimentation in this sample. Kruskal–Wallis test with post hoc Dunn's test, comparisons to the first group are shown (HP-OG488 control, no particles added). *p* < 0.05 (*), *p* < 0.01 (**), *p* < 0.0001 (****). d) The particle fractions as in (c) but for different time points and showing *n* = 25 technical repeats of *N* = 1 independent experiment each. Box plots: the center line, the median; the box limits, the upper and lower quartiles; the whiskers, minimum and maximum values.

The FCS data was analyzed as follows. First, autocorrelation curves of only labeled HP (HP-OG488) in PBS were fitted with one-component fits to obtain the characteristic diffusion time of the randomly labeled proteins. Due to the relative abundance of HSA in HP (about 60%), it is expected that these fits correspond primarily with HSA-OG488 species in solution.^[7,14]^ For the final analysis, all the autocorrelation data corresponding to the HP and incubation samples was fitted with a two-component model. One component was fixed to the diffusion time obtained for HP-OG488 above. The second component was fixed to a diffusion time characteristic for nanoparticles with a hydrodynamic diameter corresponding to the number distribution obtained from DLS measurements (Figure 2) and using the Stokes–Einstein equation. This two-component fit directly delivered the particle fraction (%) as a measure of protein binding, which was sub-sequently plotted for comparison (Figure 3). High nanoparticle fractions were obtained when high levels of protein interactions occurred, which shifted the free HP-OG488 diffusion times to mostly nanoparticle diffusion times. In the absence of protein binding, the nanoparticles were not visible by our FCS setup because they were not labeled.

Examples of normalized autocorrelation curves and two-component fits are shown in Figure 3b. A shift of the autocorrelation curves to the right indicated slower diffusion times, hence, an increase in hydrodynamic diameters. First, the difference between free OG488 (mean ± s.d.: 39.4 ± 8.0 μs, 1.2 ± 0.2 nm) and HP-OG488 (mean ± s.d.: 256 ± 34 μs, 7.8 ± 1.0 nm) confirmed successful labeling and purification of HP-OG488. The nearly perfect overlap of the HP-OG488 curve and the PEG-PBA curve means that no significant binding of HP-OG488 occurred with these unlabeled polymersomes. In contrast, the PEG-PHMA curve clearly showed two components, first for partial free HP-OG488 diffusion and a second component with slower diffusion time, corresponding to the nanoparticles, indicating protein binding. The curve for the positive control (DOTAP liposomes) was shifted much further to the right, revealing substantial aggregation in agreement with the DLS data (Figure 2).

Plotting the nanoparticle fraction percentage for all the unlabeled nanovesicle samples incubated with HP-OG488 for 24 h revealed interesting differences among the samples (Figure 3c). The DOTAP positive control showed a near-to-maximum nanoparticle fraction, confirming the suitability of this FCS technique for in situ protein fouling analysis on nanovesicles. In addition, only the gel-phase and not the liquid-phase PEGylated liposomes revealed a significant nanoparticle fraction in agreement with previous literature.^[25]^ This gel-phase formulation is based on the Doxil composition, one of the few nanomedicines on the market, and showcases clearly the formation of the human plasma-derived protein corona. This confirms the potential importance of protein corona in vivo, as found after injection and recovery of a similar product (Caelyx) in patients.^[3]^

PMOXA-polymersomes accumulated significant amounts of protein. Interestingly, the blend of the two copolymer lengths (PMOXA-mix) helped to reduce the protein amount, in agreement with our previous study on these polymersomes where we employed fetal bovine serum (FBS-OG488).^[27]^ However, unlike PEGylated liposome formulations, the protein corona formation on PMOXA-polymersomes could explain their shortened circulation time, as found in a previously reported zebrafish embryo model.^[27,51]^ When examining the time course of HP-OG488 binding (Figure 3d), it becomes evident that most nanovesicles accumulated more protein over time up to the 24 h time point. The only exception was PMOXA, the polymersome sample exposing short PMOXA chains (DP = 6) on the surface. This instant binding of random plasma components might be one reason why we previously observed worse circulation behavior of the sample PMOXA versus PMOXA-mix.^[27]^

In contrast, all the PEG-polymersomes, except PEG-PHMA, which was also unstable in 10 v/v% HP by DLS (Figure 2), did not reveal significant protein binding. Previously, PEG-based polymersomes (PEG-*b*-PBD and PEG-*b*-PDLLA) were found to circulate similarly or even longer than PEGylated liposomes when studied in mice and rats.^[31,32]^ It appears that protein corona formation might play a lesser role for PEG-polymersomes studied herein compared to PEGylated liposomes and PMOXA-polymersomes. This is also evidence of a key mechanistic difference in terms of protein binding and the corresponding downstream effects between polymersomes and liposomes, even when exposing the same polymer on the surface (PEG). There is precedence available in the literature of other PEG-based nanoparticles with long circulation behavior without the help of a protein corona.^[13]^ In addition, we tested the influence of the glass transition temperature (*T*_g_) of the hydrophobic block on the adsorption of HP-OG488. Since all the above block copolymers presented hydrophobic blocks of low *T*_g_, we synthesized poly(ethylene glycol)-*b*-poly(methyl methacrylate) (PEG-*b*-PMMA) presenting a hydrophobic block with high *T*_g_ (≈110 °C)^[52]^ (Figure S5, Supporting Information). The derived polymer assemblies presented multicompartment polymersome structures with similar sizes to the above-described library. As for the majority of PEG-polymersomes tested herein, the PEG-*b*-PMMA assemblies did not reveal significant protein binding (Figure S5, Supporting Information). After studying the full mixtures of plasma proteins binding to nanovesicles by FCS, we moved on to individual proteins to relate previously evaluated downstream effects, such as blood circulation time, to the above-found differences in protein fouling in greater detail.

### 2.3 Differences in Plasma Components Binding to Nanovesicles

**Figure 4:**
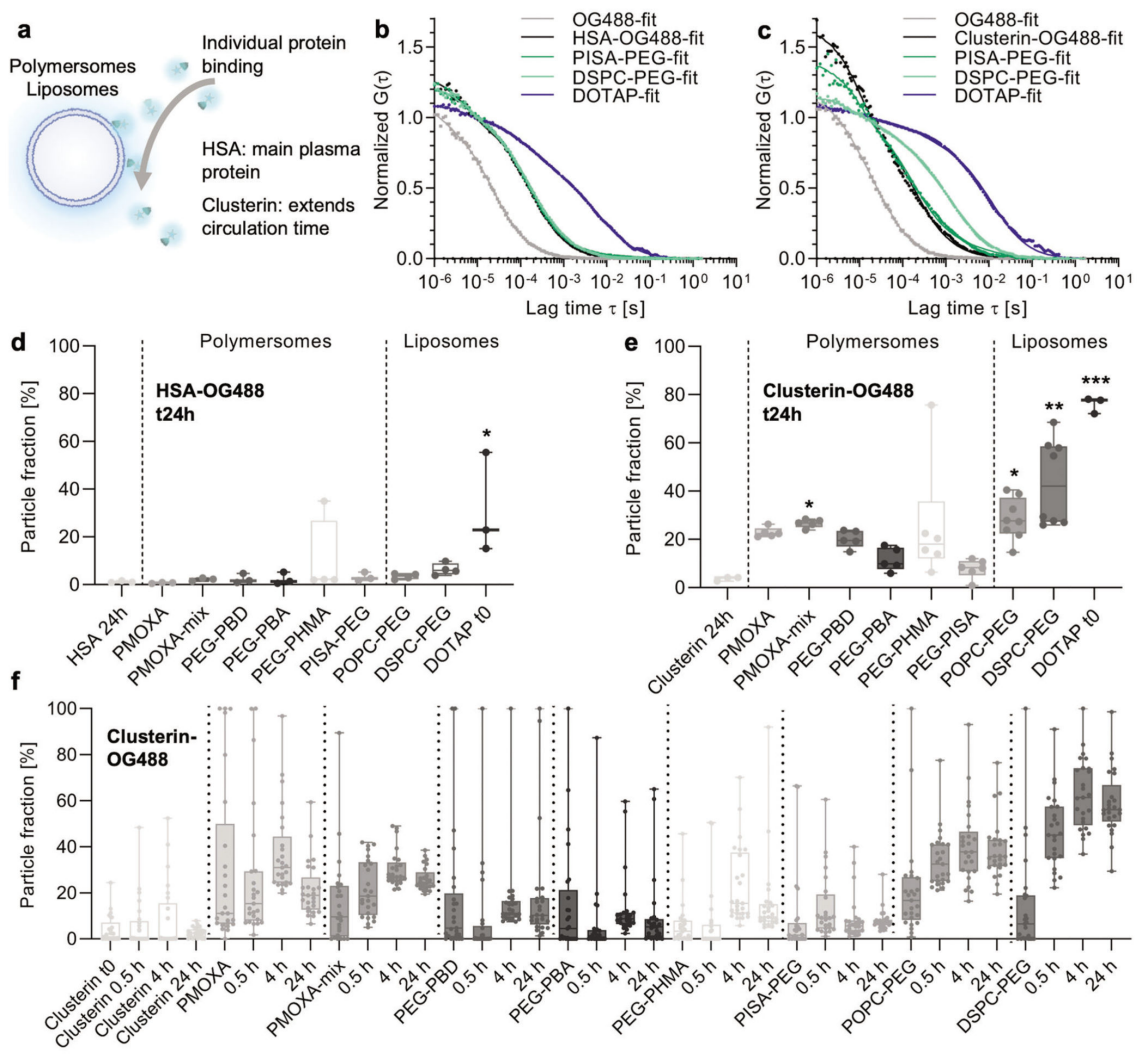
Interaction of labeled human serum albumin (HSA-OG488) and clusterin-OG488 with unlabeled polymersome and liposome samples measured by FCS. a) Schematic representation of a protein binding experiment. b,c) FCS autocorrelation curves (symbols represent raw data, solid lines are two-component fits, the average curve of *n* = 25 technical repeats, total 125 s). d,e) Particle fractions obtained from two-component fits of FCS autocorrelation curves as shown in (b,c) (see Materials and Methods in Supporting Information) for unlabeled polymersomes or liposomes incubated with either HSA-OG488 or clusterin-OG488 after 24 h of incubation at 37 °C. The experiment reveals the extent of protein binding to the nanovesicle surfaces (*N* ≥ 3 independent experiments, each dot represents the average value from *n* = 25 technical repeats). The high particle fractions represent high protein binding, while low particle fractions represent low protein binding. For the DOTAP nanovesicles, the time point at 0 h is shown, since protein addition resulted in severe aggregation and sedimentation in this sample. Kruskal–Wallis test with post hoc Dunn's test, comparisons to the first group are shown (HSA-OG488 and clusterin-OG488 controls, respectively, no particles added). *p* < 0.05 (*), *p* < 0.01 (**), *p* < 0.001 (***). f) The particle fractions as in (e) but for different time points and showing *n* = 25 technical repeats of *N* = 1 independent experiment each. Box plots: the center line, the median; the box limits, the upper and lower quartiles; the whiskers, minimum and maximum values.

Besides studying a mixture of proteins, FCS also allows the characterization of individual proteins that bind to the nanovesicles. Due to the importance of dysopsonins, such as HSA and clusterin, in reducing macrophage uptake and prolonging blood circulation time, we separately labeled these two plasma proteins in isolation and repeated the above FCS measurement performed in the previous section (Figure 4).

As in the experiments with HP (Figure 3b), the autocorrelation analysis (Figure 4b,c) shows the characteristic diffusion time shifts for some representative samples. First, successful labeling and purification of HSA-OG488 and clusterin-OG488 were confirmed. Next, the nanoparticle fraction percentage revealed no significant interaction of any of the nanovesicles with HSA-OG488, except for the positive DOTAP control (Figure 4d). In contrast to the experiments in presence of full plasma proteins (HP-OG488, Figure 3) the results highlight the importance of non-HSA plasma-derived proteins required for protein corona. We expected HSA to be the most labeled fraction of HP due to its relative abundance. However, we hypothesize that other non-labeled components may interact with HSA after first depositing on the nanovesicle and then through protein–protein interactions. We also tested clusterin (Figure 4c,e,f) as a plasma protein of low abundance (0.14% vs 60% for HSA), which has been repeatedly identified as an important dysopsonin for prolonging the blood circulation time of nanoparticles.^[7,14]^ In the case of PMOXA-based nanovesicles, PMOXA-mix bound significant amounts of clusterin. The time courses showed overall stabilization over the 24 h time period (Figure 4f). Alongside HP-OG488 revealing higher binding for PMOXA, the results suggest that PMOXA-mix could relatively adsorb more clusterin than PMOXA. This hypothesis agrees with our previous study on this nanovesicle system using FBS whereby improved circulation of PMOXA-mix in comparison to PMOXA was demonstrated.^[27]^

Interestingly, among all the tested nanovesicles, PEGylated liposomes accumulated clusterin-OG488 the most, while PEG-polymersomes repelled it. Multicompartment polymersomes based on high *T*_g_ PEG-*b*-PMMA also did not bind clusterin (Figure S5, Supporting Information). The high levels of clusterin binding on PEGylated liposomes could explain their long circulation times despite significant protein fouling (Figure 3) in agreement with PEGylated polystyrene nanoparticles.^[7]^ In the case of PEG-polymersomes, literature reports have shown similar or enhanced circulation times compared to PEGylated liposomes. For example, PEG-*b*-PBD polymersomes with varying PEG lengths showed long circulation times (τ_1/2_ from 15.8–28 h) in rats, which were similar or slightly better than PEGylated liposomes (τ_1/2_ from 10–20 h).^[31]^ In contrast, another study revealed PEG-*b*-PBD polymersomes circulation half-lives of only 117 min in healthy mice.^[33]^ Another report showcased that circulation half-lives (τ_1/2_) in mice were also longer for PEG-*b*-PDLLA polymersomes (τ_1/2_ = 47.3 h) compared to PEGylated liposomes (τ_1/2_ = 10.6 h, DPPC-Cholesterol-DSPE-PEG2k).^[32]^ In combination with literature reports, our results suggest a different mechanism for protein-nanovesicle interactions and prolonged circulation times for PEG-based polymersomes compared to liposomes and other types of PEGylated nanoparticles such as polystyrene nanoparticles.^[7]^ PEG density could be a key factor, although there is some contrasting data available in the non-vesicle literature either showcasing increased clusterin binding and reduced cell uptake with high density^[14]^ and vice versa.^[15]^ Therefore, the influence of polymersome membrane packing and dynamics, for example, lateral diffusion speed and copolymer flexibility, on protein fouling are future research directions of interest, especially since the liposome field suggests dependence of protein fouling on the phase of the lipid membrane.^[25]^ Interestingly, higher clusterin binding to PMOXA-compared to PEG-polymersomes is not associated with better circulation time, PEG-based poly-mersomes have shown better circulation times in the literature as discussed above. This can be explained by the higher random plasma binding to PMOXA (Figure 3). In contrast to most PEG-based polymersomes, PMOXA and PMOXA-mix adsorbed significant amounts of random plasma components, potentially also opsonins, which negate the effect of dysopsonin (clusterin) adsorption. Further studies on the nature of proteins adsorbing on PMOXA-based vesicles are of interest for future work. Our data also suggests that PEG-polymersomes can circulate well in the absence of significant protein binding, in agreement with a study on different, non-vesicular nanoparticles.^[13]^ Overall, this comparative study of protein fouling on various polymersomes compared to PEGylated liposomes reveals key differences between the different nanovesicle classes and important observations for moving polymersome-based nanomedicines closer to the clinic.

## 3 Conclusion

In this work, we provided a comprehensive study of the stability of various polymersomes in human plasma. Most nanovesicles were colloidally stable when incubated at 10 v/v% HP for 24 h. We further analyzed protein fouling on these nanovesicles by in situ FCS and compared all the data to PEGylated liposomes. Protein corona formation on PEGylated liposomes is key for their prolonged circulation time. We confirmed plasma protein binding to gel-phase versions mimicking marketed liposome products. The high accumulation of clusterin on these nanovesicles explains their prolonged circulation time. In contrast, PEG-polymersomes mostly did not attract significant amounts of protein in our experimental setup, highlighting a key mechanistic difference between the two nanovesicle classes. Both have been shown to provide long circulation times. However, “stealth” liposomes appear to achieve this via a protein corona, while PEG-polymersomes do so without significant amounts of protein bound to their surface. For PMOXA-polymersomes, we confirmed protein corona formation, which appeared to be detrimental in this case, as it reduced blood circulation time compared to PEGylated liposomes and PEG-polymersomes.^[27]^ Blends of copolymers with various lengths, as we have previously demonstrated for PMOXA-polymersomes,^[27]^ or mixtures of chemically different block copolymers might be interesting future avenues to tune the polymersome protein corona for specific nanomedical approaches. The observed differences between PEG-exposing polymersomes and liposomes highlight the importance of PEG density, as well as copolymer membrane flexibility and fluidity on protein fouling, warranting further studies to push polymer-somes closer toward clinical translation.

## 4 Experimental Section

All experimental details are shown in Supporting Information.

## Supplementary Material

Supplementary Materials Supporting Information is available from the Wiley Online Library or from the author.

## Figures and Tables

**Table 1:** Summary of copolymers used for polymersome self-assembly through film rehydration, solvent exchange, or photo-PISA including the degrees of polymerization (DPs).

Short names copolymers	Full copolymer names and DPs	*M*_n_[kDa]^b)^	*Ð* ^b)^
PMOXA-1^a)^	PMOXA_6_-*b*-PDMS_65_-*b*-PMOXA_6_	5.8	1.30
PMOXA-2^a)^	PMOXA_21_-*b*-PDMS_65_-*b*-PMOXA_21_	8.4	1.35
PEG-PBD^a)^	PEG_30_-*b*-PBD_47_	3.8	1.07
PEG-PBA	PEG_43_-*b*-PBA_45_	14.3	1.61
PEG-PHMA	PEG_43_-*b*-PHMA_41_	8.4	1.39
PISA-PEG	PEG_113_-*b*-PHPMA_308_	72.8	1.30

a) commercial block copolymers;

b) obtained from GPC (see Figure S1, Supporting Information).

## Data Availability

The data that support the findings of this study are available from the corresponding author upon reasonable request.
