# Supplementary material for: Differences in Human Plasma Protein Interactions between Various Polymersomes and Stealth Liposomes as Observed by Fluorescence Correlation Spectroscopy
Source: Macromol Biosci. Author manuscript; Available in PMC 2024 Jan 6. (PMC7615495; doi:10.1002/mabi.202200424)
Supplement: Supplementary Materials — Supporting Information is available from the Wiley Online Library or from the author. [file EMS175907-supplement-Supplementary_Materials.pdf]

Macromolecular Bioscience

Supporting Information

for *Macromol. Biosci.*, DOI 10.1002/mabi.202200424

Differences in Human Plasma Protein Interactions between Various Polymersomes and Stealth Liposomes as Observed by Fluorescence Correlation Spectroscopy

Adrian Najer, Omar Rifaie-Graham, Jonathan Yeow, Christopher Adrianus, Mohamed Chami and Molly M. Stevens**

Supporting Information

Differences in Human Plasma Protein Interactions Between Various Polymersomes and Stealth Liposomes as Observed by Fluorescence Correlation Spectroscopy

Adrian Najer, Omar Rifaie-Graham, Jonathan Yeow, Christopher Adrianus, Mohamed Chami, and Molly M. Stevens**

Materials and Methods

All the data was plotted using GraphPad Prism 9.0.0.

PMOXA and PEG-PBD Polymersome Formation

To form PMOXA-based polymersomes, two commercial block copolymers (PMOXA-1, Polymer Source, P18140D-MOXZDMSMOXZ (6-65-6), Mw: 500-4800-500 g/mol, *Đ* = 1.3 and PMOXA-2, P18140A-MOXZDMSMOXZ (21-65-21), Mw: 1800-4800-1800 g/mol, *Đ* = 1.35) were used according to our previous protocol.[1] Either pure 6-65-6 was used (labeled as PMOXA) or 50 mol% mixtures of 6-65-6 and 21-65-21 (labeled as PMOXA-mix). PEG-*b*-PBD (PEG-PBD, Polymer Source, P43224B-BdEO, Mw: 1300-2500 g/mol, *Đ* = 1.07) was assembled in the same way. In brief, copolymer stocks were prepared in ethanol at 20 mg/mL. Then, a thin film was formed in a glass vial by adding the desired amounts of copolymer solution and evaporating ethanol until dryness by hand using a nitrogen stream. Rehydration of the film with phosphate buffered saline (PBS, Sigma-Aldrich, D8537) followed by stirring for at least overnight at RT yielded crude polymersome solutions. These solutions were then extruded through 0.1 µm polycarbonate membranes at least 15 times (Avanti, Sigma-Aldrich, 610000). Samples were run through a PD MidiTrap column (GE Healthcare) in PBS before sterile filtration through a 0.22 µm syringe filter (Millipore, SLGV013SL). The samples were stored at 4 °C.

PEG-*b*-PBA, PEG-*b*-PHMA, and PEG-*b*-PMMA Synthesis and Polymersome Formation

The polymers were synthesized by modifying previously reported procedures.[2–5]

Synthesis of poly(ethylene glycol)-b-poly(butyl acrylate) (PEG-b-PBA): Chain extension by reversible addition-fragmentation chain transfer (RAFT) radical polymerization was performed on the macro chain-transfer agent (macroCTA) poly(ethylene glycol) 4-cyano-4-

(phenylcarbonothioylthio) pentanoate (PEG-CPADB) (M_n ~ 2000 g/mol, determined by supplier, Sigma-Aldrich) generating an amphiphilic block copolymer. The solids, AIBN (1.3 mg, 7.9 µmol, purchased from Molekula and recrystallized from methanol) and PEG-CPADB (390 mg, 0.2 mmol) were dissolved together in 0.684 mL of 1,4-dioxane (99.8%, Sigma Aldrich). In a separate container, butyl acrylate (BA) (≥99%, contains 10-60 ppm monomethyl ether hydroquinone as inhibitor, Sigma-Aldrich) was purified from its hydroquinone-based polymerization inhibitor through a basic aluminium oxide plug. Then, BA (406 mg, 0.45 mL, 3.2 mmol) was added to the AIBN and PEG-CPADB solution. The dissolved oxygen was removed by bubbling with argon for 1 h. The resulting I:CTA:M stoichiometry was 1:8:400. The reaction was initiated by transferring to a 90 °C oil bath under argon atmosphere. The reaction was quenched by exposure to atmospheric oxygen after 2 h and precipitated in a 60:40 methanol:water solution. The dispersion was centrifuged at 7000 x g for 15 min and the supernatant was discarded. To remove the non-polymerized monomer, the pellet was dissolved in 3 mL of THF (99.9%, Sigma-Aldrich) and an end of spatula of Sudan Blue II (98%, Sigma-Aldrich) was added as a small molecule indicator for preparative size exclusion chromatography (SEC). Distilled THF was employed as the mobile phase and Biobeads S-X3 (600 – 14 000 g/mol range, Bio-Rad Laboratories) as the stationary phase. The elution volume prior to the blue small molecule fraction was collected and concentrated in vacuo. Finally, the polymer was dried in a vacuum oven at 40 °C overnight and 538 mg (68 % yield) was collected.

Synthesis of poly(ethylene glycol)-b-poly(hexyl methacrylate) (PEG-b-PHMA): The same procedure was applied as for the synthesis of PEG-*b*-PBA, though in this case butyl acrylate was substituted by hexyl methacrylate (539 mg, 0.625 mL, 3.2 mmol) and the volume of 1,4-dioxane was 0.937 mL. 588 mg (63 % yield) was collected. The M_n and $Đ$ values of the described polymers are available in Figure S1 and Table 1.

Synthesis of poly(ethylene glycol)-b-poly(methyl methacrylate) (PEG-b-PMMA): The same procedure was applied as for the synthesis of PEG-*b*-PBA, though in this case the polymerization reaction consisted of AIBN (2 mg, 1.22 µmol), PEG-CPADB (195 mg, 0.2 mmol), butyl acrylate was substituted by methyl methacrylate (0.65 mL, 610 mg, 6.1 mmol) and the volume of 1,4-dioxane was 1.53 mL. The ratio I:CTA:M was 1:8:500. 531 mg (66 % yield) was collected. The M_n was found to be 15 700 g mol^{-1} and $Đ$ = 1.41 by GPC.

Self-Assembly of PEG-PBA, PEG-PHMA, and PEG-PMMA by solvent exchange: 15.1 mg of PEG-PBA, 15.3 mg of PEG-PHMA, or 10 mg of PEG-PMMA (0.95 µmol, 0.94 µmol, and 0.64 µmol, respectively) were dissolved in 0.5 mL of THF (99.9%, Sigma-Aldrich) in a 7 mL glass vial equipped with a magnetic stirrer bar. Then, 5 mL of Dulbecco's phosphate buffered saline

(PBS) (Gibco) was added at a constant rate over 3 min and the dispersions were allowed to stir for additional 10 min. The THF was allowed to evaporate to the atmospheric air overnight.

Photo-PISA synthesis of PEG-*b*-PHPMA polymersomes

The synthesis of PEG_{113}-CDTPA was performed using a protocol from literature.[6] PISA-PEG was synthesized according to a previous protocol.[7] Briefly, PEG_{113}-CDTPA (113.8 μL of a 10 mg mL^{-1} acetone stock solution, 2.11×10^{-4} mmol) was added to a 1.5 mL low protein binding microcentrifuge tube and the organic solvent was allowed to evaporate. HPMA (12.2 mg, 11.4 μL, 8.46×10^{-2} mmol) (99.7%, Sigma-Aldrich) was added, followed by 88.6 μL of 100 mM phosphate, 50 mM NaCl buffer (pH 7.4). The tube was vortexed for ~30 s and 100 μL of this solution was transferred to a Corning®, flat bottom, clear 384-well microplate. To minimize evaporation during synthesis, 20 μL of mineral oil (BioReagent, Sigma-Aldrich) was gently pipetted on top of the polymerization solution. The microplate was then immediately irradiated from above using a Teleopto LED array (λ_{max} = 405 nm, I ~ 10 mW cm^{-2}) (Bio Research Center Co., Ltd.) for 3 h. To purify the polymersome samples, the turbid solution was removed from the well, diluted to a final volume of 1 mL with PBS and spun at 16 000 × g for 10 min. The supernatant was carefully removed and the polymersome pellet resuspended in 1 mL fresh PBS. The centrifugation process was repeated an additional two times to obtain the purified PEG-*b*-PHPMA polymersomes.

Liposome Formation

Liposomes were formed using the freeze-thaw and extrusion method. The following lipids were used: 1-palmitoyl-2-oleoyl-glycero-3-phosphocholine (POPC, Avanti, 850457P-200mg), 1,2-distearoyl-sn-glycero-3-phosphocholine (DSPC, Avanti, 850365P-200mg), 1,2-dioleoyl-3-trimethylammonium-propane (DOTAP, Avanti, 890890P-200mg), 1,2-distearoyl-sn-glycero-3-phosphoethanolamine-*N*-[methoxy(polyethylene glycol)-2000] (DSPE-PEG2k, Laysan Bio, MPEG-DSPE-2000-1g), and cholesterol (Sigma-Aldrich, C8667-5G). Lipid stocks were prepared in Chloroform:MeOH 9:1 solutions. Doxil-mimicking liposomes were made from a mixture of 4.79 mg DSPC, 1.6 mg cholesterol, and 1.6 mg DSPE-PEG2k.[8] A liquid-phase version of these liposomes was made by swapping out DSPC for POPC. DOTAP-liposomes (25 wt% DOTAP) were made from 3.6 mg POPC, 1.2 mg DOTAP, and 1.6 mg cholesterol. Desired amounts of lipids were transferred to glass vials and the solvent evaporated by hand using a nitrogen stream. Films were further dried under vacuum for at least 2 h to ensure complete removal of the solvent mixture. Films were rehydrated with phosphate buffered saline

(PBS, Sigma-Aldrich, D8537) and subjected to at least three freeze-thaw cycles (-80 °C and 40 °C). These crude liposome solutions were then extruded for at least 15 times through 0.1 μm polycarbonate membranes (Avanti, Sigma-Aldrich, 610000). Samples were run through a PD MidiTrap column (GE Healthcare) in PBS before sterile filtration through a 0.22 μm syringe filter (Millipore, SLGV013SL). The samples were stored at 4 °C.

Gel Permeation Chromatography (GPC) Measurements

Polymer molecular weight (M_n, GPC) and dispersity (*Đ*) were measured using a 1260 Infinity II GPC MDS (refractive index detection only) equipped with a PSS GRAM guard column (8 x 50 mm, 10 μm) and two PSS GRAM linear columns (8 x 300 mm, 10 μm, 500-1 000 000 Da). HPLC grade DMF (containing 0.075% wt% LiBr) at 40 °C was employed as eluent at a flow rate of 1 mL/min. Molecular weight calibration was performed using near-monodisperse poly(methyl methacrylate) standards (EasiVial, Agilent).

Proton Nucleic Magnetic Resonance (^{1}H NMR) Measurements

^{1}H NMR was recorded on a JEOL 400 MHz spectrometer. All measurements were carried out in $CDCl_3$.

DLS and Zeta Potential Measurements

DLS measurements (*n* = 3) were performed on a Malvern Zetasizer Nano-ZS. 70 μL of polymersome/liposome solution in PBS was transferred into single use microcuvettes for measurement. Zeta potential measurements (*n* = 3) were performed in zeta potential cuvettes using 950 μL of 300 mM sucrose pre-mixed with 50 μL polymersome/liposome solution in PBS.

TEM and Cryo-TEM

For negative stain TEM, 5 μL of polymersome solution in PBS (0.5 mg/mL) was pipetted onto a plasma cleaned TEM grid (Electron Microscopy Sciences, CF200-Cu, 215-412-8400), which was blotted away after 1 min incubation at RT. Then, the samples were washed using two ddH_2O drops. Finally, the samples were negatively staining using two drops of 2 wt% uranyl acetate in water (0.45 μm filtered). The second drop of UA was kept on the grid for 15 s before blotting away and drying the grid overnight. TEM imaging was performed on a JEOL 2100F. For cryo-TEM, a 4 μl aliquot of sample was adsorbed onto holey carbon-coated grid (Lacey, Tedpella, USA), blotted with Whatman 1 filter paper and vitrified into liquid ethane at -180 °C using a Leica GP2 plunger (Leica microsystems, Austria). Frozen grids were transferred onto a

Talos L120C Electron microscope (FEI, USA) using a Gatan Multi-Specimen cryo-holder Model 910 (GATAN, USA). Electron micrographs were recorded at an accelerating voltage of 120 kV using a low-dose system (40 $e^{-}/Å^{2}$) and keeping the sample at -175 °C. Defocus values were -2 to 3 μm. Micrographs were recorded on 4K x 4K Ceta CMOS camera.

Human Plasma and Protein Labeling

Human plasma (HP), human serum albumin (HSA), and clusterin were all randomly labeled via coupling to free amines using a NHS-activated dye. Briefly, 25 μL HP (Biopredic, sodium heparin pooled human plasma) was added to 0.1mL phosphate buffer (0.1 M, 0.05 M NaCl, pH 7.4). In case of HSA (Sigma-Aldrich, A1659) and clusterin (2bscientific, CLU-H5227), 1 mg and 50 μg were transferred to 0.5 mL and 25 μL phosphate buffer (0.1 M, 0.05 M NaCl, pH 7.4), respectively. Next, 1.25 μL (HP), 9 μL (HSA), and 0.25 μL (clusterin) DMSO containing 0.34 mg (HP), 0.1 mg (HSA), and 25 μg (clusterin) of OG488-NHS (Invitrogen, 06149 Oregon Green™ 488 Carboxylic Acid, Succinimidyl Ester, 6-isomer) were added to the mixtures, respectively. The reactions were then incubated under shaking at room temperature for at least 3 h. Sequential size exclusion chromatography (SEC) using a PD MiniTrap (GE Healthcare) and then a PD MidiTrap (GE Healthcare) in PBS was used to purify away free OG488 from all the samples. The final stocks of HP-OG488 (equals a 1/60 dilution of HP in PBS), HSA-OG488 (0.7 mg/mL in PBS), and clusterin-OG488 (33 μg/mL in PBS) were then aliquoted and stored in the freezer at -20 °C.

FCS Measurements

Polymersome and liposome samples in PBS (all at about 2 mg/mL polymer/lipid concentration) were mixed 1:1 with a 1:10 dilution of the HP-OG488 stock in PBS (finally a 1/1200 HP dilution), with a 1:10 dilution of the HSA-OG488 stock (finally 35 μg/mL), and directly with the clusterin-OG488 stock (finally 17 μg/mL) in 0.2 mL PCR tubes. Samples were then incubated at 37 °C using a ThermoMixer at 450 RPM. 5 μL aliquots were taken from the incubation to conduct FCS measurements at each timepoint. A commercial LSM 880 (Carl Zeiss, Jena, Germany) equilibrated at 37 °C, and using a 488 nm Ar^{+} laser, appropriate filter sets, and a 40x C-Apochromat water immersion objective (NA 1.2) were used for all the FCS measurements. PyCorrfit program 1.1.6.[9] was employed to fit all the data. FCS measurements conducted on free OG488 in PBS (D = 5.49 x 10^{-6} cm^2/s at 37 °C, D = 4.1 x 10^{-6} cm^2/s at 25 °C)[10] allowed calibration of the x-y dimension of the confocal volume (ω_{xy}^2) to obtain

diffusion coefficients (D) for all the unknown samples, which were then converted to hydrodynamic diameters using Stokes-Einstein equation.

For FCS measurements, ibidi 8-well plates (80827, ibidi, Germany) were used and the laser focused 200 µm above the glass plate. For each sample, 25 intensity traces of 5 s each were recorded and autocorrelated. The autocorrelation curves shown in the paper are average curves of the full measurement series of 125 s. For analysis, HP-OG488, HSA-OG488, and clusterin-OG488 data was first fitted with one component fits $G_{1comp}(\tau)$ to yield characteristic diffusion times for the labeled proteins. Subsequently, all the data was analyzed with two-component fits $G_{2comp}(\tau)$. The diffusion time τ_1 of the first component was fixed to the diffusion time obtained above for free protein diffusion. The second component was fixed to diffusion times characteristic for nanoparticles with a hydrodynamic diameter as obtained from the DLS number distributions for all the different nanovesicle samples (τ_2). Due to extensive aggregation of DOTAP vesicles, the second component (τ_2) was not fixed for this sample and only t0 and the first 5 traces could be used for analysis due to fast sedimentation. For correction of dyes moving into the triplet state, a triplet fraction T with corresponding triplet time τ_{trip} was added and fixed between 1 – 10 µs. The structural parameter SP was set to a fixed value of 5. These two-component fits then yielded the fractions with corresponding diffusion times (F_1, F_2; $N = n1 + n2$). F_2 *100 represents the % of nanoparticles, hence, the amount of protein binding, which was used in all the plots.

$$G_{1comp}(\tau) = \left(1 + \frac{T}{1-T} e^{\frac{-\tau}{\tau_{trip}}}\right) * \frac{1}{N * \left(1 + \frac{\tau}{\tau_D}\right) * \sqrt{1 + \frac{\tau}{SP^2 \tau_D}}}$$

$$G_{2comp}(\tau) = \left(1 + \frac{T}{1-T} e^{\frac{-\tau}{\tau_{trip}}}\right) * \frac{1}{N} * \left[\frac{F_1}{\left(1 + \frac{\tau}{\tau_1}\right) * \sqrt{1 + \frac{\tau}{SP^2 \tau_1}}} + \frac{1 - F_1}{\left(1 + \frac{\tau}{\tau_2}\right) * \sqrt{1 + \frac{\tau}{SP^2 \tau_2}}}\right]$$

At least two independent samples were prepared for each vesicle type and at least three independent experiments (*N* = 3) were then conducted for all the protein binding studies (all with *n* = 25 technical repeats).

Statistical Analysis

Sample sizes and employed statistical tests and post hoc analysis are explained in all the figure captions. Data normalization and representation is clarified in each caption. GraphPad Prism 9.0.0 was used to analyze and plot all the data.

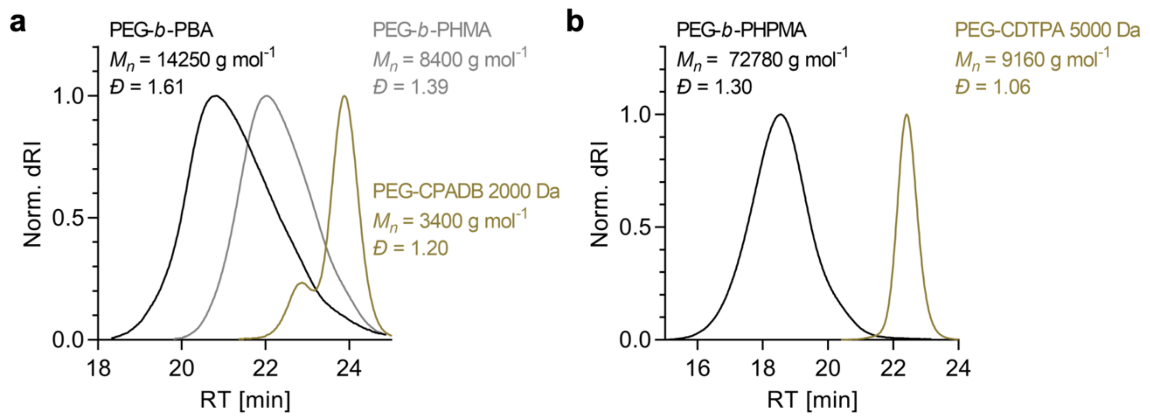

Figure S1. GPC analysis of the synthesized block copolymers. (a) PEG-*b*-PBA and PEG-*b*-PHMA in comparison to the macro-RAFT agent PEG-CPADB. (b) PEG-*b*-PHPMA in comparison to the macro-RAFT agent PEG-CDTPA.

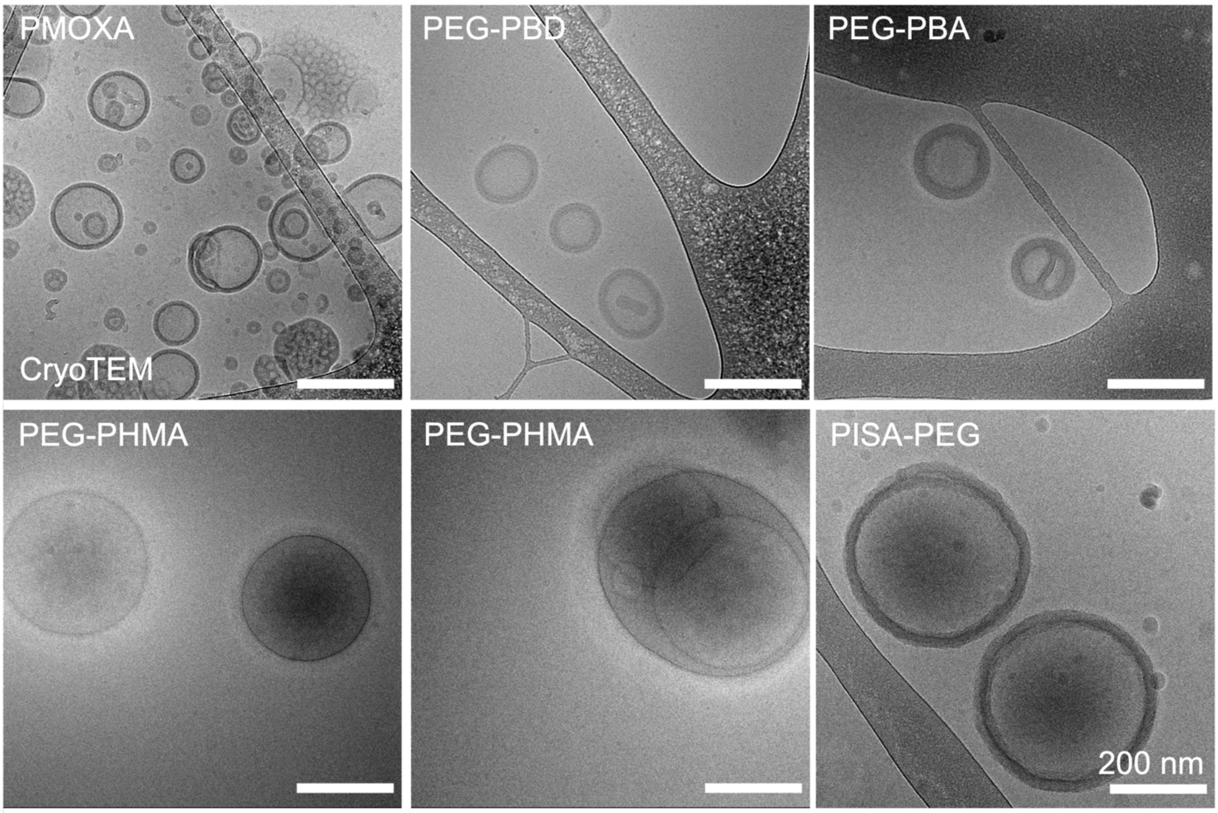

Figure S2. Additional cryo-TEM images for Figure 1 from the main text. Scale bars, 200 nm.

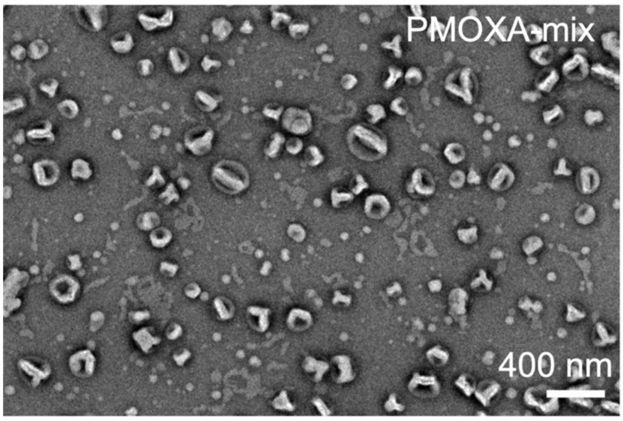

Figure S3. Overview negative TEM image of PMOXA-mix. Scale bar, 400 nm.

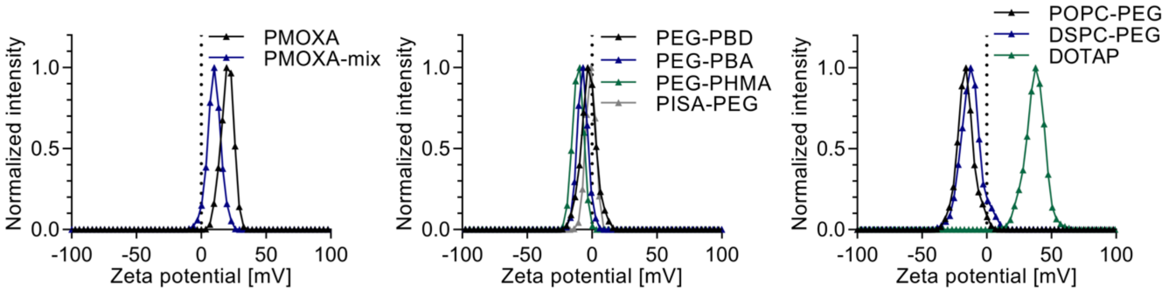

Figure S4. Zeta potential curves for all vesicle types. Mean values are given in main Figure 1.

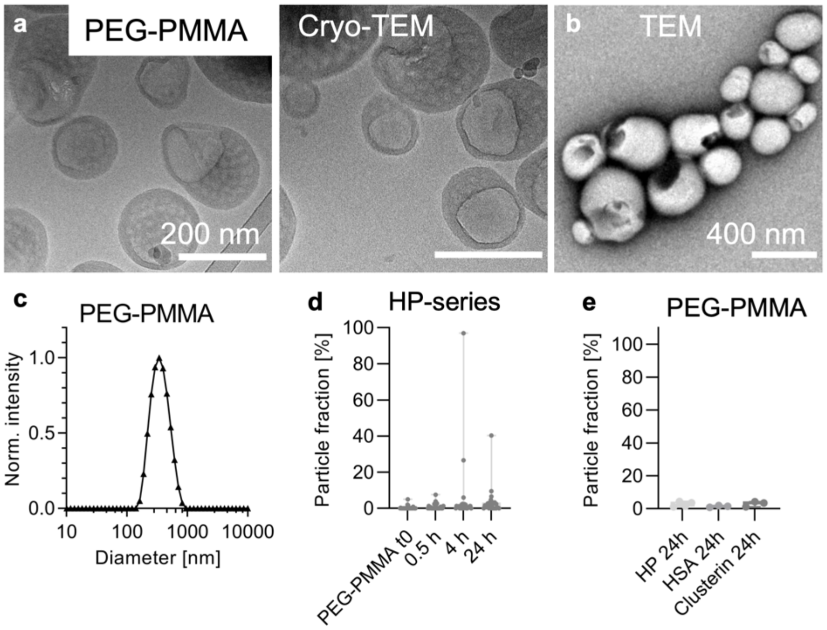

Figure S5. (a) Cryo-TEM and (b) negatively-stained TEM images of PEG-*b*-PMMA assemblies. Scale bars 200 nm (a) and 400 nm (b), respectively. (c) DLS intensity distribution for PEG-*b*-PMMA assemblies in PBS (mean of technical triplicates). (d) Particle fractions obtained from two-component fits of FCS autocorrelation curves for unlabeled PEG-*b*-PMMA assemblies mixed with HP-OG488 after incubation at 37 °C. ($N = 1$ independent experiment, $n = 25$ technical repeats). e) Particle fractions obtained from two-component fits of FCS autocorrelation curves for unlabeled PEG-*b*-PMMA assemblies mixed with either HP-OG488,

HSA-OG488 or clusterin-OG488 after 24 h of incubation at 37 °C. ($N \geq 3$ independent experiments, each dot represents average value from $n = 25$ technical repeats).

References

[1] A. Najer, A. Belessiotis-Richards, H. Kim, C. Saunders, F. Fenaroli, C. Adrianus, J. Che, R. L. Tonkin, H. Høgset, S. Lörcher, M. Penna, S. G. Higgins, W. Meier, I. Yarovsky, M. M. Stevens, *Small* **2022**, *18*, 2201993.
[2] O. Rifaie-Graham, S. Ulrich, N. F. B. Galensowske, S. Balog, M. Chami, D. Rentsch, J. R. Hemmer, J. Read De Alaniz, L. F. Boesel, N. Bruns, *J Am Chem Soc* **2018**, *140*, 8027.
[3] O. Rifaie-Graham, N. F. B. Galensowske, C. Dean, J. Pollard, S. Balog, M. G. Gouveia, M. Chami, A. Vian, E. Amstad, M. Lattuada, N. Bruns, *Angewandte Chemie - International Edition* **2021**, *60*, 904.
[4] M. Rodriguez-Perdigon, S. Jimaja, L. Haeni, N. Bruns, B. Rothen-Rutishauser, C. Rüegg, *Macromol Biosci* **2022**, *22*, 2200168.
[5] O. Rifaie-Graham, J. Yeow, A. Najer, R. Wang, R. Sun, K. Zhou, T. Dell, C. Adrianus, C. Thanapongpibul, S. Mann, J. Read de Alaniz, M. M. Stevens, *Nat. Chem. Accepted* **2022**.
[6] S. Xu, J. Yeow, C. Boyer, *ACS Macro Lett* **2018**, *7*, 1376.
[7] H. Kim, J. Yeow, A. Najer, W. Kit-Anan, R. Wang, O. Rifaie-Graham, C. Thanapongpibul, M. M. Stevens, *Advanced Science* **2022**, 2200239.
[8] A. Soundararajan, A. Bao, W. T. Phillips, R. Perez, B. A. Goins, *Nucl Med Biol* **2009**, *36*, 515.
[9] P. Müller, P. Schwille, T. Weidemann, *Bioinformatics* **2014**, *30*, 2532.
[10] P. Kapusta, P. GmbH, **2010**, 0.
